# Scorpionism in Pará, Brazil: Clinical assessment of neuromuscular manifestations

**DOI:** 10.1590/0037-8682-0053-2025

**Published:** 2025-08-08

**Authors:** Rosicléia Freitas Borges, Joyce Nascimento Dergan, Pasesa Pascuala Quispe Torrez, Vera Lúcia Teixeira de Freitas, Maria Rita Bertolozzi, Átila Barros Magalhães, Fernanda Jacqueline Teixeira Cardoso, Rogério Bertani, Francisco Oscar de Siqueira França

**Affiliations:** 1 Secretaria Municipal de Saúde de Rurópolis, PA, Brasil.; 2 Universidade Estadual do Pará, Santarém, PA, Brasil.; 3 Hospital de Clínicas Dr. Radamés Nardini - Fundação ABC, Santo André, SP, Brasil.; 4 Departamento de Infectologia e Medicina Tropical, Faculdade de Medicina, Universidade de São Paulo, São Paulo, SP, Brasil.; 5 Laboratório de Investigação Médica em Imunologia (LIM48) do Hospital das Clínicas da Faculdade de Medicina, Universidade de São Paulo, São Paulo, SP, Brasil.; 6 Departamento de Enfermagem em Saúde Coletiva, Escola de Enfermagem, Universidade de São Paulo, São Paulo, SP, Brasil.; 7 Laboratório Especial de Ecologia e Evolução, Instituto Butantan, São Paulo, SP, Brasil.; 8 Núcleo de Medicina Tropical do Departamento de Infectologia e Medicina Tropical, Faculdade de Medicina, Universidade de São Paulo, São Paulo, SP, Brasil.

**Keywords:** Scorpionism, Scorpion sting, Envenomation, Severity assessment, Neuromuscular manifestations, Amazonia

## Abstract

**Background:**

In the western region of Pará (Brazil), the clinical manifestations of scorpionism exhibit unique characteristics, and there is currently no proposal for a quantitative assessment of this condition. This manuscript proposes and applies a classification system for assessing the severity of scorpionism, comparing it with the system used by the Ministry of Health.

**Methods:**

This quantitative, descriptive, observational, cross-sectional study evaluated victims of scorpionism treated at the Municipal Hospital of Rurópolis in Pará, Brazil. Clinical and therapeutic data were collected using instruments and scales, particularly the International Cooperative Scale for the Evaluation of Ataxias.

**Results:**

Thirty-four patients were assessed between January and July 2023. The majority were young males. All presented with pain, an 'electric shock sensation' and myoclonus. Muscle spasms were observed in 65% of patients, postural changes in 64%, dysmetria in 55%, altered eye movements in 50%, and dysarthria in 44%. While these manifestations are transitory, they cause significant discomfort to the patient. The classification proposed in this study was compared to the Ministry of Health's Severity Classification, and low agreement between the two classifications was shown (only in 50% of cases). The proposed classification raised the severity of 17 patients by one degree: fifteen from mild to moderate and two from moderate to severe, one of whom developed rhabdomyolysis and acute kidney injury.

**Conclusions:**

The proposed classification was beneficial and could be used in the future to assess the effectiveness of therapeutic interventions for this frequent and neglected condition in the Amazon.

## INTRODUCTION

There are approximately 2.200 recognized species of scorpions worldwide, 104 of which are of medical importance^1^ ^,^ ^2^. Scorpion envenomation is common in tropical and subtropical regions^3^.

Brazil is home to approximately 160 scorpion species, and scorpionism is a growing, albeit neglected, public health challenge associated with significantly increasing morbidity and mortality rates. In 2023, 201,690 cases of scorpion envenomation and 153 deaths were reported in Brazil. In Pará state, which is located in northern Brazil, 3,618 cases of scorpion envenomation and three deaths were reported this year^4^. 

Scorpions in the genus *Tityus* are found throughout Brazil and are primarily responsible for cases of human envenomation. Among the *Tityus* scorpions, *T. serrulatus* is the most common cause of severe scorpion envenomation and death. *T. bahiensis* and *T. serrulatus* are found in most regions in Brazil, *T. stigmurus* is predominantly found in the northeast, and *T. obscurus* is found in the northern region, mainly in the states of Pará and Amapá^5^ ^-^ ^8^.

Despite the relative frequency of scorpion stings in the north, scorpion species that can cause unique clinical manifestations, which have not been reported in other regions of Brazil or the world, have been found in some areas of the Brazilian Amazon, especially in the western part of Pará State^9^ ^-^ ^14^.

The common clinical manifestations of scorpionism include pain, hypertension or hypotension, cardiac arrhythmias, sweating, nausea, and vomiting^6^ ^,^ ^15^ ^,^ ^16^. Severe cases often lead to acute heart failure, acute pulmonary edema, shock, and, ultimately, death^6^ ^,^ ^17^.

The treatment of scorpion envenomation depends on the severity of the clinical manifestations. Pain-relief medication is typically used for mild cases, whereas antivenom therapy is necessary for moderate-to-severe cases. The Brazilian Health System provides antivenom treatment for free, and the treatment involves the administration of anti-scorpion serum (SAEEs) or anti-arachnid serum (SAAr)^18^. Notably, the latest Note No. 25 from the Ministry of Health, which was adapted to include treatment of scorpionism does not address envenomation in some regions of the Amazon^19^.

Unlike the typical clinical presentations of scorpion envenomation reported in other parts of Brazil and the world, which usually include manifestations consistent with involvement of the sympathetic and parasympathetic autonomic nervous systems, scorpionism in certain regions of the Brazilian Amazon, particularly western Pará and neighboring areas, has distinct manifestations. Affected patients in this area mainly present with intense pain, “electric shock” sensations, muscular disorders, acute cerebellar dysfunction, and other nervous system-related symptoms^9^ ^,^ ^11^ ^-^ ^14^ ^,^ ^20^. Varying degrees of some of these clinical manifestations have been observed in patients stung by *T. obscurus*, *T. strandi*, and *T. apiacas*, however, other *Tityus* species may also be involved in these cases^9^ ^,^ ^11^ ^-^ ^14^.

Given the unique nature of these envenomations and the lack of a comprehensive and systematic classification system for the clinical manifestations observed in affected patients, we proposed guidelines for assessing and classifying the severity of scorpion envenomation in these regions and applied it in this research. The classification is based on an objective evaluation of the most characteristic clinical manifestations of these envenomations and an extensive review of the existing literature and the results of prospective observational studies^9^ ^,^ ^11^ ^,^ ^21^. The four tests in the International Cooperative Ataxia Rating Scale (ICARS), which are used for identifying the signs of acute cerebellar dysfunction, were adapted and condensed for this classification^21^ ^-^ ^23^.

This classification includes the application of standardized scales and can be performed in any healthcare unit. It involves the evaluation of ocular changes, speech disorders, kinetic dysfunction, and standing or walking ability (Table 1). The scales are easy to use and do not require any laboratory tests^21^. However, it is important to emphasize that envenomation in patients who presents with manifestations of intense acute cerebellar dysfunction, even a few hours after a scorpion sting, and considered severe in ICARS Scale, should be defined as moderate because this clinical picture do not pose a risk to the patient's life as they progressive and regress rapidly, generally on the second day after envenomation.

**TABLE 1:** Neuromuscular symptoms of scorpionism in the Brazilian Amazon: proposed classification for assessment of severity.

Classification	Clinical Manifestations
Mild	Presence of pain and/or paresthesia, local or regional “electric shock” sensation.
	Heat, edema, erythema, sweating, and piloerection may occur at the sting site.
Moderate	May present with any of the manifestations described for mild cases plus one or more of the following manifestations:
	Electric shock sensation in one half of the body or throughout the body.
	Abnormal muscle movements (altered muscle tone, myoclonus, hypertonia or hypotonia, fasciculation, spasm) without rhabdomyolysis.
	Changes in the eyes (I), speech (II), kinetic functions (III), and posture or gait (IV) based on International Cooperative Ataxia Rating Scale (ICARS) scores, regardless of the intensity*.
	·Vomiting may occur.
Severe	Any manifestations described for the mild and moderate cases plus one or more of the following:
	Manifestations compatible with rhabdomyolysis such as intense hypertonia, frequent spasms, and increased serum muscle enzyme levels such as creatine kinase, aldolase, lactic dehydrogenase and myoglobinuria.
	Any evidence of acute kidney injury: choluria, oliguria, increased serum urea, and creatinine levels.
	Intense gastrointestinal manifestations such as incoercible vomiting.
	Marked change in blood pressure, cardiac arrhythmia, intense agitation, convulsion.
*International Cooperative Ataxia Rating Scale (ICARS) scores	
**I. Changes in eye tracking** (the patient is asked to follow the slow, lateral movement made by the examiner's finger) **or the presence of nystagmus**.	
Minimal	0: Normal
Moderate	1: Slightly saccadic
Intense	2: Clearly saccadic
**II. Speech disorders (dysarthria)** (The patient is asked to repeat a standard sentence several times, always the same, e.g. The rat gnawed the King of Rome’s clothes**)**	
Minimal	0: Normal
Moderate	1: Mild change in fluency
	2: Moderate change in fluency
	3: Considerable slowness and dysarthric speech
Intense	4: Speechless
**III. Alterations in kinetic function - Index-nose test** (decomposition and dysmetria). (The patient rests their hand on their knee before starting the movement. Visual control is required. Three repetitions with each limb must be performed for proper assessment)	
Minimal	0: Without difficulty
Moderate	1: Oscillating motion without decomposition
	2: Segmented movement in two phases and/or moderate dysmetria when reaching for the nose
	3: Segmented movement in more than two phases and/or considerable dysmetria when reaching for the nose
Intense	4: Dysmetria prevents the patient from reaching the nose
**IV. Changes in posture or gait - Ability to stand with eyes open (ataxia)** (The patient is first asked to try standing on one foot. If this is impossible, the patient should try to stand with both feet in tandem. If this is not possible, the patient should stand with both feet together. In the natural position, the patient is asked to find a comfortable standing position).	
Minimal	0: Normal: able to stand on one foot for more than 10 seconds
	1: Able to stand in tandem, but unable to stand on just one foot for more than 10 seconds
Moderate	2: Able to stand with feet together, but unable to stand with feet in tandem
	3: Unable to stand with feet together, but able to stand in the natural position without support, without swaying, or with moderate swaying
	4: Able to stand in the natural position without support, but with considerable oscillations and corrections
Intense	5: Unable to stand in the natural position without the firm support of an arm. If there is a risk of the patient falling or feeling uncomfortable, the test is suspended
	6: Completely unable to stand, even with firm support from both arms. Due to the patient's limitations, this item will not be tested

However, in the rare cases in which patients present with marked continuous hypertonicity and spasms that progress to striated muscle lesions with rhabdomyolysis should be considered severe. Destruction of skeletal muscle releases myoglobin into circulation, resulting in myoglobinuria and increasing the risk of progression to acute kidney injury, which, if not adequately treated, can lead to death^24^.

The aim of this study was to use evaluation scales to classify the severity of this peculiar scorpion envenomation according to the presence of some clinical characteristics in a region of western Pará or in any other region where these clinical manifestations are observed.

## METHODS

This was cross-sectional observational study was conducted at the Municipal Health Services (MHS) in Rurópolis, Pará, Brazil.

### Study setting

**FIGURE 1:**
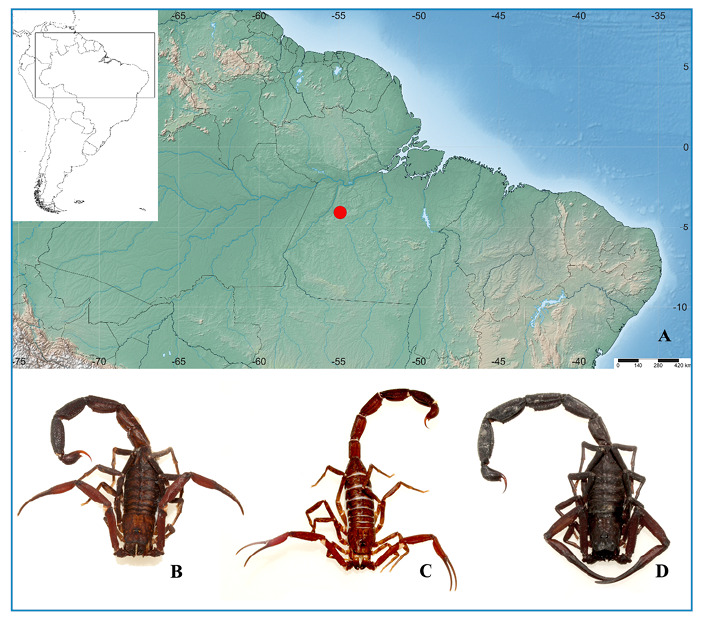
**(A)** Rurópolis municipality- SimpleMappr (Shorthouse 2010). **(B)** Male *T. obscuru*s, the causative species in Case n° 01. **(C)** Male *T. obscurus*, causative species in Case n° 09. **(D)** Male *T. obscurus*, causative species in Case n° 21. All patients were from a rural area (Rurópolis, 2023). *Laboratório Especial de Ecologia e Evolução, Instituto Butantan, São Paulo, 2023.*

Rurópolis municipality is located in the south-west mesoregion of Pará, along the BR-230 Trans-Amazon Highway and at the junction of the BR-163 Santarem-Cuiabá Highway, approximately 1130 km from the state capital, Belém (Latitude: -4.08839, Longitude: -54.9142 4° 5′ 18″ South, 54° 54′ 51″ West) (Figure 1A).

The local economy relies on family-based agriculture and extensive livestock farming. There are five health centers in the municipality (Rurópolis Municipal Hospital [RMH] and four basic rural health units) that treat patients with scorpion stings.

### Study participants

This study included 34 individuals who were stung by scorpions and visited the Emergency Department at the RMH between January and July 2023. Patients with cognitive deficits, children younger than 12 years old, those who did not agree to participate, and those who were discharged or left the hospital before the researchers could assess them were excluded.

### Ethical considerations

All the patients signed an informed consent form after receiving comprehensive information about the study. This study was conducted in accordance with the guidelines outlined in Resolution 466/12 of the Brazilian National Health Council. The study was approved by the Research Ethics Committee of the University of São Paulo Nursing School (protocol number: 5.508.668, 2022).

### Data forms

A form was used to obtain and systematically collate patient data. Measurement tools were used to evaluate patient symptoms; however, this evaluation was discontinued if the patient exhibited some discomfort or risk. The tests were organized in increasing order of complexity, allowing the researcher to observe the difficulty level of each test for each patient. 

The assessments were performed within approximately 30 minutes after patient admission, ensuring that the necessary patient care was not disrupted.

During the stay at the RMH, the healthcare team provided care to each patient, including admission, recording of medical history, clinical assessment, drug treatment, administration of antivenom (if necessary), and mandatory reporting. 

In cases where the scorpion that stung the patient was captured and delivered to the health center, a specialist from Butantan Institute was contacted for identification of the species.

### Clinical assessments

The severity of patients' conditions was assessed using two instruments. The first was based on guidelines outlined by the Ministry of Health guidelines^19^ and was administered by attending physicians. The second was the protocol proposed in this study (Table 1).

The proposed protocol involves the use of specific scales adapted for clinical assessment of neurological manifestations, particularly motor and sensory manifestations (Table 1). The ICARS is commonly used for evaluation of limitations associated with cerebellar dysfunction and has been validated for use in Brazilian populations^21^ ^-^ ^23^ ^,^ ^25^ ^-^ ^27^.

The visual analog scale (VAS) was used for the assessment of pain (Table 1)^28^.

We found no assessment tool for “electric shock” sensation. Therefore, we created a scale for this study after testing previously in several patients (Table 1).

Muscle tone was assessed using the modified Ashworth Scale and by evaluating the presence of myoclonus and spasms (Table 1)^29^. Cranial nerve function, level of consciousness, and reflexes were also assessed using routine neurological examination tests.

### Data analysis

The data collected in this study were organized in an Excel spreadsheet, coded, and analyzed descriptively.

## RESULTS

Thirty-four patients with scorpionism from Rurópolis municipality were included in this study (Figure 1A). 

Scorpion envenomation was more prevalent in males (64.7%), especially in those aged 20-49 years, which represents an economically active age group. Most of the patients had not completed primary school (64.7%), and 88.2% reported that they are Brown. Urban and rural residences distribution was very similar; however, most envenomations (76.5%) occurred in rural areas (Supplementary Table 1). 

Three patients brought the scorpions with them, and all were identified as *T. obscurus* (Figures 1B, C, and D ). 

### Clinical description

All 34 patients presented with at least one local clinical manifestation, whereas 24 (70.6%) presented with systemic manifestations (Supplementary Table 2). 

Fifty percent of the patients reported pain and a “hot” sensation at the sting site, edema was observed in 41% of the patients, and four patients reported a localized electric shock sensation. The most prevalent systemic manifestations were myoclonus, electric shock sensation, postural alterations, dysmetria, ocular alterations, pain, and dysarthria. Notably, an electric shock sensation was reported by all patients, local in 4 (15.3%) patients and regional or systemic in 22 (84.6%) patients.

### Evaluation of clinical manifestations using scales

Pain, electric shock sensation, and myoclonus characteristics were assessed using the scales listed in Table 2.

**TABLE 2:** Characterization of pain, electrical shock sensation, and myoclonus in patients with scorpionism, Rurópolis (Pará) municipality, January to July 2023.

Pain (Visual Analogue Scale - VAS)		
Intensity	N=34	%
Painless (0)	2	5.9
Mild (1 and 2)	1	2.9
Uncomfortable (3 and 4)	4	11.8
Painful (5 and 6)	4	11.8
Horrible (7 and 8)	8	23.5
Martyrdom (9 and 10)	15	44.1
**Location**	**N=32**	**%**
Sting site	16	50.0
Entire body	8	25.0
Affected limb	6	18.8
Side of body	2	6.2
**Interval between sting and onset of pain**	**N=32**	**%**
up to 30 min	29	90.7
> 30 min - 1h	1	3.1
1 - 3 h	1	3.1
3 - 6 h	1	3.1
**Duration**	**N=32**	**%**
> 1h	3	9.4
1 - 3h	7	21.9
3 - 6h	1	3.1
6 - 12h	8	25.0
12 - 24h	11	34.4
> 24h	2	6.2
**Electrical shock sensation (in this study)**		
**Location**	**N=26**	**%**
Entire body	21	80.8
Sting site	4	15.4
Limb affected	1	3.8
**Interval between sting and onset of electrical shock**	**N=26**	**%**
Up to 30 min	6	23.1
1 - 3 h	17	65.4
3 - 6 h	2	7.7
6 - 12 h	1	3.8
**Duration**	**N=26**	**%**
< 1h	1	3.8
1 - 3h	1	3.8
3 - 6h	3	11.5
6 - 12h	10	38.5
12 - 24h	9	34.6
>24h	2	7.7
**Shock progression**	**N=26**	**%**
Increasing	25	96.1
Immediate	1	3.8
Other shock characteristics	N=26	%
Intermittent	22	84.6
Occurs after stimuli	20	76.9
Skin hypersensitivity	10	38.5
**Myoclonus (Modified Ashworth Scale)**		
**Location**	**N=20**	**%**
Entire body	16	80.0
Sting site	1	5.0
Side of body	1	5.0
Limb affected	1	5.0
Abdomen	1	5.0

**n/NT:** Cases observed/total analyzed.

Thirty-two patients reported pain. Sixteen (50%) reported that the pain was not limited to the envenomation site, 29 (90%) stated that the pain started within 30 minutes, 21 (65.63%) reported that the pain lasted for more than 6 hours, and 23 (71.88%) reported that the pain was intense (Table 2).

All 26 patients evaluated reported electric shock sensation. Twenty-one (80.77%) patients stated that this sensation extended to the entire body, whereas one (3.9%) patient reported that the sensation was localized in the affected limb. Twenty-three (88.5%) reported that the sensation began within three hours of the sting, whereas 20 (77%) patients stated that they felt the sensation after stimulation (auditory and/or light and/or tactile).

Myoclonus (local or systemic) was observed in all 20 patients and was present throughout the body in 16 (80.0%) patients (Table 2).

Supplementary Table 3 shows the results of the assessment of some cranial nerve functions, consciousness level (Glasgow Scale), tendon reflexes, degree of muscle strength, and muscle tone. Regarding the cranial nerve function assessments, 15 patients presented with altered eye movements, one presented with impaired mastication, and facial mimicry was abnormal in 11 (32.3%) patients, suggesting functional impairment of some cranial nerve pairs. 

Assessment of consciousness level indicated that only 4 (11.76%) patients exhibited altered eye-opening after verbal stimulation; the rest exhibited normal consciousness levels. Tendon reflexes were assessed in 28 patients and 15 (43.5%) showed alterations. Of the 30 patients who underwent evaluation of muscle strength, only seven (23.3%) exhibited decreased strength; the remaining patients demonstrated normal strength. Of the 27 patients who underwent assessment of muscle tone, 11 (40.7%) showed increased muscle tone.

Cerebellar function-related manifestations, including the presence of oculomotor disorders (nystagmus), speech disorders (dysarthria), kinetic functions (dysmetria-index-nose maneuver), and posture disorders, were assessed using the ICARS Scale adapted for the patients. Any alteration in these manifestations confirmed the presence of cerebellar dysfunction (Table 3).

**TABLE 3:** Assessment of neuromuscular manifestations of scorpionism (according to the ICARS scale) in Rurópolis (Pará) municipality, between January and July 2023.

ICARS Scale	*n/N_T_	%
Oculomotor disorder		
0 (Normal)	15/30	50.0
1 (Slightly saccadic)	4/30	13.3
2 (Clearly saccadic)	11/30	36.7
Dysarthria		
0: Normal	19/34	55.9
1: Mild fluency modification	4/34	11.8
2: Moderate fluency modification	4/34	11.8
3: Considerable slowing and dysarthric speech	6/34	17.7
4: Speechless	1/34	2.9
**Standing with eyes open**		
0: Normal: able to stand on one foot for more than 10 seconds	12/33	36.4
1: Able to stand in tandem, but unable to stand on one foot for more than 10 seconds	7/33	21.2
2: Able to stand́ with feet together, but unable to stand́ with feet in tandem	0/33	0.0
3: Unable to stand with feet together, but able to stand in the natural position without support, without swaying, or with moderate swaying	2/33	6.1
4: Stands in the natural position without support, with considerable swaying and correction	3/33	9.1
5: Unable to stand in the natural position without the firm support of an arm	1/33	3.0
6: Completely unable to stand, even with firm support from both arms.	8/33	24.2
**Index maneuver - Nose**		
0: No difficulty	15/33	45.5
1: Oscillating movement without decomposition	5/33	15.2
2: Segmented movement in two phases and/or moderate dysmetria when reaching the nose	7/33	21.2
3: Segmented movement in more than two phases and/or considerable dysmetria when reaching the nose	3/33	9.1
4: Dysmetria prevents the patient from reaching the nose	3/33	9.1
**Presence of muscle spasms**	**(N=34)**	**%**
0: No spasms	12/34	35.3
1: Only spasms precipitated by stimuli	2/34	5.9
2: Spontaneous spasms, less than one spasm per hour	0/34	0.0
3: Spontaneous spasms, one or more spasms per hour	2/34	5.9
4: Spontaneous spasms, more than 10 spasms per hour	18/34	52.9

*n/NT: Cases observed/total analyzed.

Oculomotor disturbances (saccadic movement) were observed in 15 (50.0%) of the 30 patients assessed. One patient had eyelid ptosis and another had nystagmus. Dysarthria was assessed in all 34 patients, and speech disorders were identified in 15 (44.2%). Evaluation of postural disorders, which included assessment of the ability to stand with the eyes open, revealed alterations in 21 patients (66.3%). Most of these patients were unable to stand independently, even with support (Table 3). 

Two tests were performed to assess kinetic functions. In the index-nose test, which assesses movement decomposition and dysmetria, changes in limb movements were observed in 18 patients (54.5%) (Table 3). The results of the index-index test were practically the same as those of the index-nose test. 

Twenty-two patients (64.7%) exhibited muscle spasms, whereas 18 (52.9%) had spontaneous spasms (Table 3).

### Severity assessment and treatment

Upon admission, patients were assessed for symptom severity, and specific treatment plans were developed. The doctor in charge classified most cases (23; 67.7%) as mild and 11 (32.3%) as moderate (Table 4). Notably, 13 (38.2%) patients received a variable number of SAEE vials. 

**TABLE 4:** Correlation between the severity classification in the Ministry of Health Guidelines and the protocol proposed in this research conducted in Rurópolis (Pará) municipality, January to July 2023.

		Ministry of Health Protocol			Total
		Mild	Moderate	Severe	
Proposed protocol	Mild	8	0	0	8
	Moderate	15	9	0	24
	Severe	0	2	0	2
Total		23	11	0	34

Gray highlights cases of agreement between the two classifications.

Categorization of the cases into grades I, II and III, which represent mild, moderate, and severe symptoms, respectively, showed that the criteria proposed in this study increased the severity of 17 cases by one grade (15 from mild to moderate and 2 from moderate to severe). One case of a 22-year-old male patient who received three vials of SAEE was classified as severe according to the proposed classification. The patient was discharged on request and returned the following day with oliguria and later with anuria. Tests performed at the RMH one day after admission revealed the following findings: urea, 93 mg/dL; creatinine, 2.1 mg/dL; urine, 1; and hemoglobin, +++. The results obtained the following day were as follows: blood glucose, 188 mg/dL; urea, 115 mg/dl; creatinine, 2.3 mg/dl; and creatine kinase (CK), 2.991 U/L. The patient’s condition deteriorated rapidly to acute kidney injury and he was transferred to a referral hospital where he underwent hemodialysis.

## DISCUSSION

To our knowledge, this is the first study conducted to document the use of quantitative assessment scales for the evaluation of unique clinical manifestations of scorpionism in Amazonian regions, mainly sensory and neuromotor impairments, particularly acute cerebellar dysfunction. The results of this study demonstrated that these scales can provide objective data on the clinical severity of scorpionism. 

Seventy-seven cases of envenomations were reported in the study region between January and July 2023, 34 (45.4%) of which were included in this study^4^. The epidemiological data of the patients included in this study are similar to those of patients from other regions and areas of the country^8^ ^,^ ^9^ ^,^ ^11^.

People injured by venomous animals do not usually take the animal that caused the envenomation to the health center. All the three specimens obtained in the present study were *T. obscurus*. We attributed these cases to *T. obscurus* because of the high prevalence of this species in the region and the characteristic clinical features of envenomation caused by this species. However, envenomation by another scorpion species in the region, *T. strandi*, can have a similar clinical picture^13^. Nevertheless, given the rarity of *T. strandi* in the region, it is unlikely that cases of envenomation caused by this species were included in the study sample.

Contrary to observations in other regions of Brazil, more than 75% of the envenomations analyzed in the present study occurred in rural areas. The environmental degradation caused by deforestation and fires in the study region can render a rural environment conducive to scorpion proliferation owing to accumulation of organic matter, garbage, and debris, which attracts insects, a scorpion food source. This scenario is similar to what is observed in cities^7^ ^,^ ^30^.

In this study, the upper limbs, especially the hand, were the most affected anatomical site (23 patients; 67.65 %). Similar results have been reported by Pardal et al. (2003)^9^.

Regarding the interval between the time of the sting and hospital admission, most patients were able to reach the health center within three hours. However, some patients visited the health center between 6 and 9 hours after the sting due to the late appearance of the most uncomfortable symptoms. In addition, the delay in seeking care may be attributable to difficulty traveling from more distant communities, which is the reality of the Amazon region. Notably, delay in receiving care can worsen clinical conditions^31^ ^,^ ^32^.

Local manifestations recorded in other studies showed similar results^9^ ^-^ ^12^. In a study conducted in Santarém (PA), local symptoms were observed in 91.7% of patients and systemic manifestations in 98.6%, which are higher than the frequencies observed in the present study. Neurological manifestations were more common than autonomic and general manifestations in the present study, with the most common symptom being an electric shock sensation throughout the body, affecting 88.9% of the patients. The most common symptoms in the previous study were myoclonus (93%), dysmetria (86.1%), dysarthria (80.6%) and gait ataxia (70.8%)^9^.

In another study conducted in Santarém (PA), pain was the most frequent local manifestation, affecting 46 patients (79.2%). Other local manifestations such as edema and erythema have been described less frequently. Systemic symptoms appeared within an average of 15 minutes after the scorpion sting, and they were described as an “electric shock sensation” in almost all cases (94.8%). The most prevalent neuromuscular signs were myoclonus (n=44, 75.9%), ataxia (n=42, 72.4%), dysarthria (n=36, 62%), dysmetria (n=31, 53.4%), fasciculations (n=27, 46.5%), hyperreflexia (n=16, 27.6%), restlessness (n=14, 24.1%), drowsiness (n=12, 20.7%), and nystagmus (n=2, 3.4%)^11^. Notably, the severities of these clinical symptoms were not assessed in these tsudies^9^ ^,^ ^11^. Additionally, spontaneous muscle movements of varying amplitudes were frequently reported. 

These systemic manifestations observed in the present study and the two above mentioned studies conducted in the western region of Pará are different from those obserwved in cases reported in other regions of the country, where autonomic nervous system manifestations are predominant^6^ ^,^ ^18^.

Patients with cerebellar dysfunction generally present with slurred speech; that is, slow speech with complex articulation of words and variable voice volume control^33^. This manifestation was observed more frequently in the other studies conducted in Western Pará, which were published in 2003 (80.2%) and 2015 (62%)^9^ ^,^ ^11^. 

Posture disorders were also reported in these two regional studies, with gait ataxia observed in 70.8% of patients of the study published in 2003 and 62% in of patients of the study published 2015^9^ ^,^ ^11^. It is important to highlight that an inability to walk increases the risk of falls^9^ ^-^ ^11^. Notably, ataxia is a physical examination finding typically associated with impaired cerebellar function^33^.

Regarding kinetic function (assessed using the index-nose and index-index tests), we observed impairments in 18 patients (54.6 %). In the 2003 study^9^, a high frequency of dysmetria was observed in 62 (86.1%) of 72 cases of envenomations. The results reported in the 2015 study are similar to the results of the present study^10^ ^-^ ^11^.

Muscle spasms, which occurred in almost two-thirds of the patients in the present study, decreased in both intensity and frequency over time. Spasms can be spontaneous or occur after tactile stimulation, and can last for several days in a few cases.

It should be noted that these neuromuscular manifestations can be very uncomfortable and debilitating due to their intensity, frequency, simultaneity, and multiplicity, limiting and even preventing patients from performing basic activities such as walking and eating for long periods, lasting up to days in some cases. Impairment of cerebellar and muscular functions poses potential risks to patient safety, especially by increasing the risk of falls. In addition, continuous hypertonia for hours and the occurrence of spasms can result in rhabdomyolysis, and if severe enough, acute kidney injury^11^ ^,^ ^24^ ^,^ ^34^.

Pain, a characteristic manifestation of scorpion stings, including those of medical importance, occurs early in almost all cases of scorpionism, often spreads to other parts of the body, and is typically intense and long lasting^6^ ^,^ ^18^ ^,^ ^19^.

The study revealed that “electric shock” sensation is a characteristic symptom of scorpion envenomation, with a relatively early onset (within three hours after the sting). The sensation usually radiates outwards and increases in intensity over time (for more than six hours). In addition, it can be intermittent and triggered by stimuli. The results presented in our study provide detailed information on the characteristics of this unique manifestation of scorpion envenomation in this region of the Amazon. The identification of this characteristic symptom is fundamental for differentiating envenomations in the Amazon region from those caused by scorpion species in other regions of Brazil^6^ ^,^ ^9^ ^,^ ^11^ ^,^ ^18^.

In this study, 20 patients exhibited myoclonus, which also appears to be a characteristic of envenomation in this region^9^ ^,^ ^11^. However, this manifestation regressed within two days in most cases.

The scales used for patient assessment in the present study allowed for careful evaluation of the intensity and duration of clinical manifestations. In particular, the adapted and simplified ICARS Scale can be used to identify and quantify various cerebellar dysfunctions characteristics of this envenomation. These instruments, which have not been used in other studies, demonstrate the originality of this research and may be used for more accurate patient assessment in cases of scorpion envenomation as well careful quantitative evaluation of therapeutic interventions.

There are no local, regional, or national guidelines for assessing the severity or dosage of SAEEs for the treatment of this scorpion envenomations. This explains the difficulty in establishing the appropriate dose of SAEEs, according to the severity of envenomation, in this envenomation, in this region of the Amazon or elsewhere.

Although anti-scorpion and anti-arachnid therapies are considered the only specific treatments for scorpion stings, Torrez et al.^11^ did not find an antivenom derived from the *T. serrulatus* venom effective for controlling neurological and muscular symptoms. This outcome is expected because the venom components of *T. obscurus* are not antigenically similar to those of *T. serrulatus* ^15^. The results of the present study demonstrate that an electric shock sensation is a very early and almost universal symptom of this envenomation, caused by some species in the study region, and precedes cerebellar and muscular manifestations.

Rhabdomyolysis may be associated with severe cases of scorpionism, which can progress to acute kidney injury. In this situation, the severity of skeletal muscle damage can be evaluated using creatine kinase levels^11^. 

The main limitation of this study is the time at which the scales were administered. The assessments were conducted at different times to minimize patient discomfort. In addition, all patients were assessed after receiving initial care and symptomatic therapy. Therefore, the scales may not fully reflect the maximum neuromuscular impairment experienced by the patients after envenomation. Another limitation is that the inclusion of only hospitalized patients may have introduced some selection bias as patients with mild cases are typically not hospitalized. So, the patients included in this study may have had a more serious clinical condition than the 77 cases reported in Rurópolis during the research period.

The results of the quantitative evaluations conducted in the present study and findings from previously published studies indicate that the clinical picture of scorpion envenomations have a broad clinical spectrum, with some patients presenting with only discreet manifestations, such as pain and “electric shock” sensation, only in the sting region, and others presenting with multiple neurological manifestations, including cerebellar function impairments and multiple abnormal movements.
