# Supplementary material for: Scorpionism in Pará, Brazil: Clinical assessment of neuromuscular manifestations
Source: Rev Soc Bras Med Trop. 2025 Aug 8;58:e0053-2025. doi: 10.1590/0037-8682-0053-2025 (PMC12333616; doi:10.1590/0037-8682-0053-2025)
Supplement: Supplementary file 1 [file 1678-9849-rsbmt-58-e0053-2025-supp1.pdf]

SUPPLEMENTARY TABLE 1: Description of the socio-demographic profile of the victims of scorpionism, Rurópolis (Pará) municipality, January to July 2023.

Socio-demographic profile	n/N_T	%
**1. Sex**		
Male	22/34	64.7
**2. Age range**		
12 to 19	4/34	11.8
20 to 49	23/34	67.6
>50	7/34	20.6
**3. Ethnic group**		
Brown	30/34	88.2
Black	3/34	8.8
White	1/34	2.9
**4. Educational level**		
Incomplete Elementary School	22/34	64.7
Complete Elementary School	3/34	8.8
Incomplete High School	4/34	11.8
Complete High School	3/34	8.8
University	2/34	5.9
**5. Home area**		
Rural	18/34	52.9
Urban	16/34	47.1
**6. Area where the accident happened**		
Rural	26/34	76.5
Urban	8/34	23.5

n/NT: Cases observed/total analyzed.
