# Supplementary material for: Scorpionism in Pará, Brazil: Clinical assessment of neuromuscular manifestations
Source: Rev Soc Bras Med Trop. 2025 Aug 8;58:e0053-2025. doi: 10.1590/0037-8682-0053-2025 (PMC12333616; doi:10.1590/0037-8682-0053-2025)
Supplement: Supplementary file 2 [file 1678-9849-rsbmt-58-e0053-2025-supp2.pdf]

SUPPLEMENTARY TABLE 2: Description of the clinical manifestations of scorpion envenomations in 34 people in the municipality of Rurópolis (Pará) between January and July 2023.

Clinical manifestations	*n/N_T	%
**1. Local events**		
Local heat	17/34	50.0
Pain	16/32	50.0
Edema	14/34	41.2
Electric shock	4/26	11.8
Erythema	2/34	5.9
Myoclonus	1/20	2.9
**2. Systemic events**		
Myoclonus	19/20	95.0
Electric shock sensation	22/26	84.6
Posture: standing with eyes open	21/33	63.6
Dysmetria	18/34	54.6
Oculomotor disorder	15/30	50.0
Pain	16/32	50.0
Dysarthria	15/34	44.1
Nausea	5/34	14.7
Hypertension	5/34	14.7
Sweating	4/34	11.8
Sialorrhoea	3/34	8.8
Tremors	3/34	8.8
Feeling of sand in the eyes	3/34	8.8
Dysphagia	2/34	5.9
Headache	2/34	5.9
Vomiting	1/34	2.9
Eyelid ptosis	1/34	2.9
Nystagmus	1/34	2.9
Dyspnoea	1/34	2.9

***n/NT:** Cases observed/total analyzed.
