# Supplementary material for: Scorpionism in Pará, Brazil: Clinical assessment of neuromuscular manifestations
Source: Rev Soc Bras Med Trop. 2025 Aug 8;58:e0053-2025. doi: 10.1590/0037-8682-0053-2025 (PMC12333616; doi:10.1590/0037-8682-0053-2025)
Supplement: Supplementary file 3 [file 1678-9849-rsbmt-58-e0053-2025-supp3.pdf]

SUPPLEMENTARY TABLE 3: Assessment of cranial nerves, level of consciousness, tendon reflexes, degree of muscle strength and muscle tone in victims of scorpionism in the municipality of Rurópolis (Pará), January to July 2023.

Neuromuscular Assessment Scales	% (n/N)		
Cranial Pairs Assessment	Eye Movement	Chewing	Facial expression
Altered	50.0 (15/30)	3.0 (1/33)	34.4 (11/32)
Preserved	50.0 (15/30)	97.0 (32/33)	65.6 (21/32)
**Assessment of Level of Consciousness (Glasgow Scale)**			
Score 15	88.2 (30/34)		
Score 14	11.8 (3/34)		
**Tendon reflex assessment**			
Decreased	7.1 (2/28)		
Normal	46.4 (13/28)		
Alive	39.3 (11/28)		
Exalted	7.1 (2/28)		
Evaluation of the degree of muscle strength	(30)		
Grade 5: Normal strength against the examiner's resistance	76.7 (23/30)		
Grade 4: Reduced muscle strength against examiner resistance	13.3 (4/30)		
Grade 3: Patient overcomes gravity, but cannot move against examiner resistance	3.3 (1/30)		
Grade 2: Movement only if resistance from gravity is removed	6.7 (2/30)		
Grade 1: Only outline of movement or fasciculations	0.0 (0/30)		
Grade 0: No movement	0.0 (0/30)		
**Assessment of muscle tone - modified Ashwort scale**			
0: No increase in muscle tone.	59.3 (16/27)		
1: Slight increase in muscle tone, manifested as resisting and yielding or minimal resistance in the extension of passive movement.	22.2 (6/27)		
1+: Slight increase in muscle tone, manifested as a slight resistance during the rest of the movement, less than half.	7.4 (2/27)		
2: More marked increase in tone during most of the extension of the movement, but with ease of movement of the affected segment.	11.1 (3/27)		
3: Considerable increase in muscle tone, with difficulty in passive movement.	0.0 (0/27)		

n/NT: Cases observed/total analyzed.
